# Supplementary material for: Association between oral microbiome and seven types of cancers in East Asian population: a two-sample Mendelian randomization analysis
Source: Front Mol Biosci. 2023 Nov 21;10:1327893. doi: 10.3389/fmolb.2023.1327893 (PMC10702768; doi:10.3389/fmolb.2023.1327893)
Supplement: Supplementary file 1 [file DataSheet1.ZIP › Supplementary Materials/MR plots of saliva/saliva═╝/ovarian cancer/pheno.2920_to_ovarian cancer_forest.pdf]

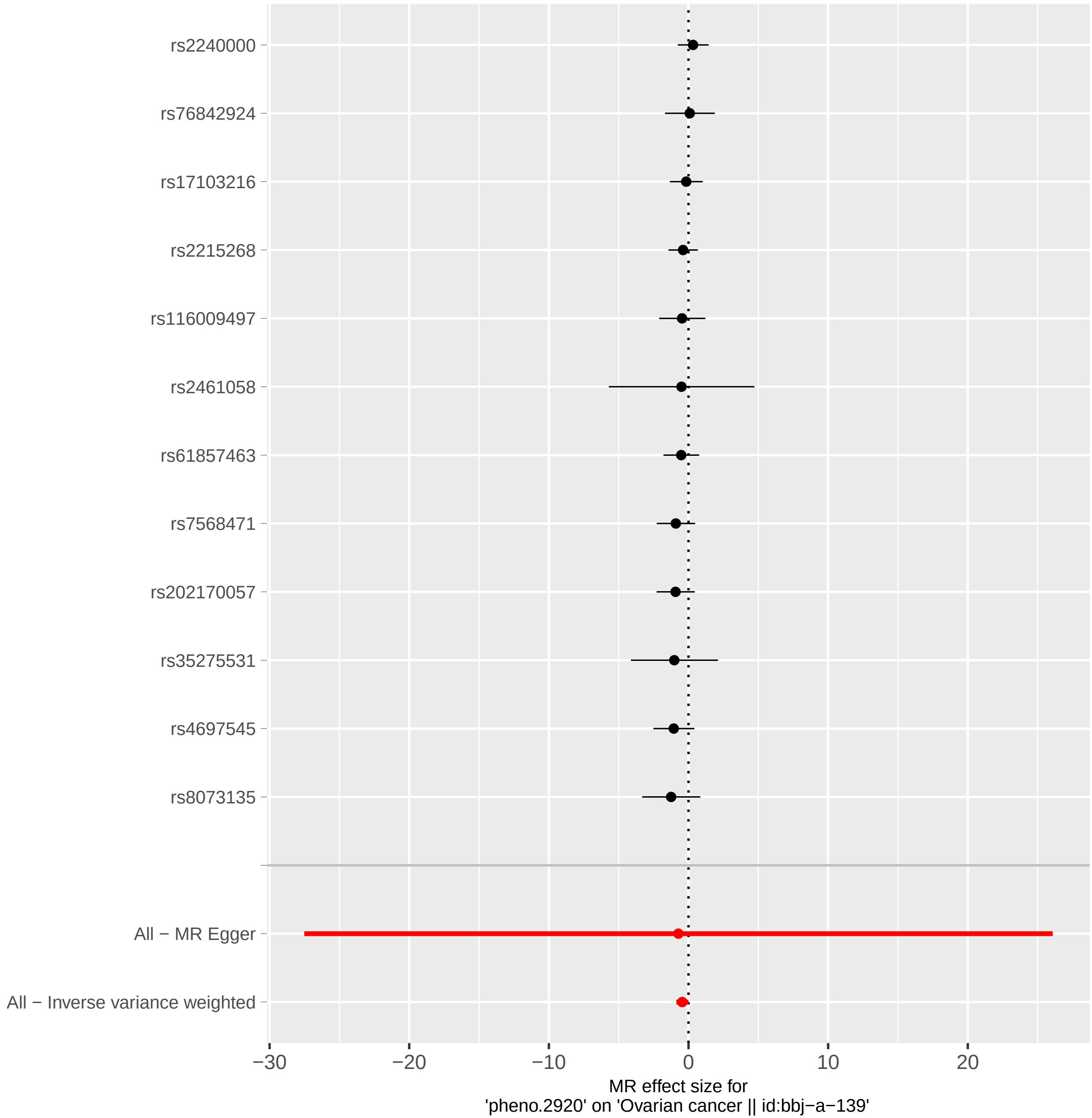
rs2240000
rs76842924
rs17103216
rs2215268
rs116009497
rs2461058
rs61857463
rs7568471
rs202170057
rs35275531
rs4697545
rs8073135
All − MR Egger
All − Inverse variance weighted
−30
−20
−10
0
10
20
MR effect size for
'pheno.2920' on 'Ovarian cancer || id:bbj−a−139'
